# Classical Forecasting of International Tourist Arrivals to Thailand

**DOI:** 10.1007/s44199-022-00041-5

**Published:** 2022-03-19

**Authors:** Sukanya Intarapak, Thidaporn Supapakorn, Witchanee Vuthipongse

**Affiliations:** 1 Department of Mathematics, Faculty of Science, Srinakharinwirot University, Bangkok, 10110 Thailand; 2 Department of Statistics, Faculty of Science, Kasetsart University, Bangkok, 10900 Thailand; 3 Department of Tourism, Ministry of Tourism and Sports, Bangkok, 10210 Thailand

**Keywords:** Foreign tourist, Forecasting, Box–Jenkins, Decomposition, Exponential smoothing, Mean absolute percentage error, Root mean square error

## Abstract

The objectives of this work are to find the suitable forecasting model and forecasting period of the number of foreign tourists traveling to Thailand. The monthly data is gathered during January 2008 to December 2019 and is divided into two sets. The first set is the data from January 2008 to December 2018 for the modelling by the method of decomposition, Holt–Winter’s exponential smoothing method and the Box–Jenkins. The second is the monthly data in 2019 for comparing the performance of the forecasting models via the criteria of the lowest mean absolute percentage error (MAPE) and the root mean square error (RMSE). The results show that, in term of forecasting, the multiplicative decomposition is the most accurate technique for the short-term (3 months) forecasting period with the lowest MAPE and RMSE of 1.04% and 42,054.29 international tourists, respectively.

## Introduction

**Fig. 1:**
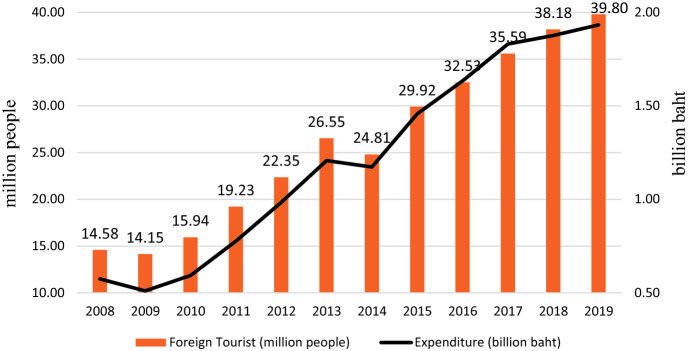
The international tourist arrivals (in million people) and their expenditures (in billion baht) in 2008–2019

The kingdom of Thailand is located in the center of mainland Southeast Asia. Thailand shares the border with Myanmar and Laos to the north, with Laos and Cambodia to the east, with the Gulf of Thailand and Malaysia to the south and with the Andaman Sea and Myanmar to the west. There are 76 provinces and Bangkok is the capital. The popular provinces are Phuket, Krabi, Chiang Mai, Nan, Buriram, Maehongson, Ayutthaya also Bangkok. Thailand encompasses diverse ecosystems, cultures, also, religious backgrounds. This diversity enchants tourists both Thais and foreigners. The famous tourist attractions are, for example, beaches, islands, floating markets, temples, mountains, waterfalls, national park, full moon parties and civilization of the cities. According to the data from [1], Fig. 1 clearly displays the increasing trend of both the number of foreign tourists (in million people) entering to Thailand and their expenditures (in billion baht). The percentage increase of foreign tourist arrivals of 2019 to 2008 is 172.88%. This may be due to the political factors in Thailand that are likely to improve which affect investors’ confidence, the recovery of the economy of Thailand and other countries in the world, organizing an illegal tour, the low-cost airlines, the more direct flight, or even an increase in inflation rate according to oil prices.

Tourism becomes one of the significant sectors that drives the economic growth of Thailand. It can be seen that the income from the tourism industry varies with the number of foreign tourists entering Thailand. The Thai government realizes the importance of the tourism industry as evidenced by the tourism action plan of the Tourism Authority of Thailand (TAT) which has four goals [2]. One of those is aiming to be the popular tourist destination for sustainability and still generating the highest tourism income in the world top 7. Hence, there are several strategies to build the international market, i.e., adjusting the image of the Thai tourism brand, boosting consumer spending, and expanding the market base of special interests such as medial and wellness tourism, sport Tourism, marriage celebration, wedding anniversary including traveling to visit relatives.

Due to the COVID-19 outbreak situation, countries all over the world have implemented travel restrictions to control the outbreak within the country. For the impact of the COVID-19 outbreak on international travel, the World Tourism Organization (UNWTO) has reported that in the first quarter of 2020, the world's international tourism has declined 22% and is expected for the full year of 2020 to drop by 60% from the previous year. In Thailand, the pursuant to the Declaration of Emergency Situations in all areas of the Kingdom of Thailand starting on 26 March 2020 including the announcement of the Civil Aviation Authority of Thailand (CAAT) to temporarily ban the inbound passenger flights result in no foreign tourists traveling to Thailand since April throughout September 2020 [1, 3].

Tourism is one of the significant contributors to Thailand’s economy. Moreover, the tourist numbers help in understanding the tourism story. The purpose of this paper is to forecast the number of foreign tourists entering Thailand which is very important for the policy planning of the country's economy. The pre-COVID-19 data of the number of tourist arrivals to Thailand is considered to analyze under normal circumstance. In order to achieve the research goal, three classical forecasting methods: decomposition, Holt–Winter’s exponential smoothing and the Box–Jenkins, are applied to get the estimates and the details will be stated in the next section.

## Literature Review

Time series forecasting is an important topic in practical application including business and industry, government, economics, environmental sciences, medicine, social science, politics, and finance [4]. Several forecasting approaches of modeling are employed to get accurate forecasts based on previous data. In 2012, Tularam, Wong and Nejad [5] analyzed tourist arrivals to Australia using time series analysis by an autoregressive integrated moving average (ARIMA) and Vector Auto-Regression models using Australian tourist arrival data (1956–2010). The results showed that ARIMA(2,2,2) performed better in term of the prediction in 2010.

In 2013, Keerativibool [6] applied the methods of Box–Jenkins, Winters’ multiplicative exponential smoothing, decomposition, and combined forecasting to forecast the number of international tourist arrivals to Thailand. The combined forecasting method was the most powerful in terms of forecasting the next 6 monthly values based on the criterion of maximum correlation coefficient between the actual data and the forecasts followed by the decomposition, Winters’ multiplicative exponential smoothing, and Box–Jenkins, respectively. Later, in 2014, Saothayanun et al. [7] compared the methods of Box–Jenkins and Holt–Winters’ multiplicative for forecasting the number of international tourists to Thailand. Base on the criteria of the root mean square error and mean absolute percent error of the forecasting values, the Winters’ method performed better than the Box–Jenkins.

In 2017, Subedi [8] suggested the alternative approach of modeling combining the autoregressive model with polynomial (biquadratic) function on time series data with monthly/seasonal fluctuation to forecast the number of tourist arrivals in Nepal. The trend was represented by the autoregressive part and the monthly fluctuation/seasons was done by biquadratic part. Recently, Roshan and Jahufer [9] implemented the Holt–Winters’ Method and Seasonal Autoregressive Integrated Moving Average (SARIMA) method to forecast the tourist arrivals in Sri Lanka. The results revealed that SARIMA provided the least root mean square error and mean absolute deviation.

Recently, Xie et al. [10] proposed the decomposition-ensemble approach to enhance the predictive accuracy of tourism demand forecasting of Hong Kong from nine sources: mainland China, Korea, Japan, the USA, Philippines Singapore, Australia, the UK, and Thailand. The competing models are the naïve, ARIMA and the artificial neural network (ANN) models. Hwandee and Phumchusri [11] aimed to forecast the international tourist arrivals to Thailand from 5 major countries: mainland China, Malaysia, Korea, and Russia during Jan 2013 to September 2018. In terms of the mean absolute percentage error, the seasonal autoregressive integrated moving average outperformed the multiple regression model with several important economic factors: income, price, exchange rate, and seasonal effect. Additionally, Rahman and Lee [12] presented the artificial neural network forecasting with the missing values imputation. The accuracy performance was validated by the mean absolute error and root mean square error of two forecasting methods which are SARIMA and ANN. Based on the imputation method of the decomposition, SARIMA performed better. In the other way, based on the imputation method of the spatial weighting method, ANN outperformed. Lately, in 2021, Janjua et al. [13] adopted the univariate time series forecasting method of tourist arrival based on the data from January 1991 to March 2020. The ARIMA(12, 1, 12) was suggested to forecast the international arrival from April 2020 to December 2020. The forecasts showed that Thailand will face significant negative zone arrival of international tourists due to the COVID-19 pandemic crises, which adversely affect Thailand’s economic due to the shortfall of international tourist arrivals.

From the forecasting methods mentioned above, different models have been applied and developed for tourism demand forecasting. They corporate the classical approach [5–13], the machine learning approach [11, 13], and the combination of classical approaches and/or the machine learning approaches [6, 8, 9, 11, 13]. The objective of this paper is to apply three classical forecasting methods: decomposition, Holt–Winter’s exponential smoothing and the Box–Jenkins to get the estimate of the tourism demand base on the monthly historical time series from 2008 to 2019. The remainder of this paper is organized as follows. Section 3 describes the data sets, the classical forecasting methods; decomposition, Holt–Winters’ exponential smoothing and Box–Jenkins, and the performance evaluation based on the root mean square error and mean absolute percent error. The results of the time series forecasting analysis are in Sect. 4. Section 5 is finally the conclusions and discussion.

## Methodology

### Data Sets

**Fig. 2:**
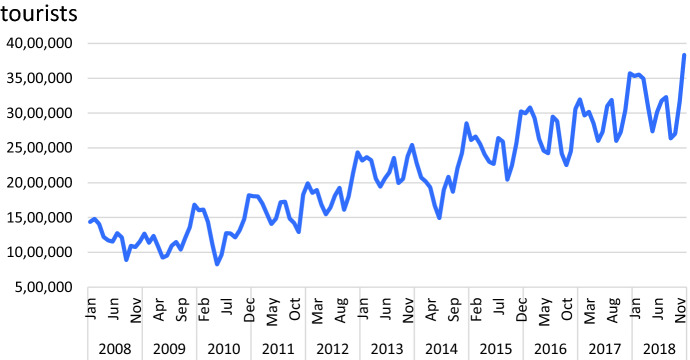
Time series plot of international tourist arrivals (in million people) from January 2008 to December 2018

The monthly data of the foreign tourist arrivals to Thailand from 2008 to 2019 [1] is divided into two segments—the fitting and the forecasting. The fitting data segment from January 2008 to December 2018 is for constructing the time series modeling and displayed in Fig. 2. The forecasting segment contains the monthly data in 2019 is for forecasting and competing forecasting models.

### Forecasting Methods

#### Decomposition

The classical decomposition approach [4] is to break the time series (*Y*_*t*_) down into the component parts of trend (*T*_*t*_) and season (*S*_*t*_). Typically, the model of decomposition is categorized as the additive model

$$Y_{t} = T_{t} + S_{t} + \varepsilon_{t}$$

and multiplicative model

$$Y_{t} = T_{t} S_{t} \varepsilon_{t}$$

where $\varepsilon_{t}$ is the random error.

The trend component is estimated using the simple linear regression model with the least squares estimates. The seasonal factor is calculated for each period in the season by detrended time series. Herein, the multiplicative model of the decomposition method is considered because the amplitude of the seasonal fluctuations varies with the level of the series as displayed in Fig. 2.

#### Holt–Winters’ Exponential Smoothing

The forecasting equation of (Holt) Winters’ multiplicative exponential smoothing [14], an extension of Holt’s method, is

$$\hat{Y}_{t + p} = ( {L_{t} + pT_{t} } )S_{t - s + p} ,$$

where, $L_{t} = \alpha \frac{{Y_{t} }}{{S_{t - s} }} + ( {1 - \alpha } )( {L_{t - 1} + T_{t - 1} } )$ is the exponentially smooth series or level estimate, $T_{t} = \beta ( {L_{t} - L_{t - 1} } ) + ( {1 - \beta } )T_{t - 1}$ is the trend estimate, $S_{t} = \gamma \frac{{Y_{t} }}{{L_{t} }} + ( {1 - \gamma } )S_{t - s}$ is the seasonality estimate, $\alpha$ is smooth constant for the level, $\beta$ is smooth constant for the trend estimate, $\gamma$ is smooth constant for seasonality estimate, *p* is the period to be forecast into the future and $0\,\, \le \alpha ,\,\,\beta ,\,\,\gamma \le \,\,1$.

#### Box–Jenkins

The Box–Jenkins approach [14] uses an iterative model-building strategy that consists of selecting an initial model (model identification), estimating the model coefficients (parameter estimation), and analyzing the residuals (model checking). The model fits well if the residuals ae generally small, randomly distributed and contain no useful information.

The general class of models, representing time series containing seasonal fluctuations, is a seasonal ARIMA process. It is formed by including additional seasonal terms in the ARIMA, that is, SARIMA(p,d,q) (P,D,Q)_s_ defined as follows

$$\phi_{p} ( B )\Phi_{P} ( B )( {1 - B} )^{d} ( {1 - B^{s} } )^{D} Y_{t} = \phi_{0} + \omega_{q} ( B )\Omega_{Q} ( {B^{s} } )\varepsilon_{t}$$

where $( {1 - B^{s} } )Y_{t} = Y_{t} - Y_{t - s}$, *d* is the order of differencing, *s* is the number of seasons per year, and *D* is the order of seasonal differencing. The operator polynomials are

$$\phi_{p} ( B ) = ( {1 - \phi_{1} B - \ldots - \phi_{p} B^{p} } )$$

$$\omega_{q} ( B ) = ( {1 - \omega_{1} B - \ldots - \omega_{q} B^{q} } )$$

$$\Phi_{P} ( {B^{s} } ) = ( {1 - \Phi_{1} B^{s} - \ldots - \Phi_{P} B^{sP} } )$$

$$\Omega_{Q} ( {B^{s} } ) = ( {1 - \Omega_{1} B^{s} - \ldots - \Omega_{Q} B^{sQ} } ).$$

In addition, the residuals should be independent and normally distributed.

### Measures of Accuracy

#### Mean Absolute Percentage Error (MAPE)

The mean absolute percentage error [14] is computed by finding the absolute error in each period, dividing by the actual observed value for that period, and then averaging these absolute percentage errors, that is, $MAPE = \frac{1}{n}\sum\nolimits_{t = 1}^{n} {\frac{{| {Y_{t} - \hat{Y}_{t} } |}}{{Y_{t} }}} \times 100$.

#### Root Mean Squared Error (RMSE)

The RMSE [14] is the square root of the mean squared error which is the sum of the squared residuals and divided by the number of the observations. The unit of RMSE is the same as the original series and RMSE is given by $RMSE = \sqrt {\frac{1}{n}\sum\nolimits_{t = 1}^{n} {( {Y_{t} - \hat{Y}_{t} } )^{2} } }$.

## Results

### Forecasting Methods

#### Decomposition

Based on the multiplicative decomposition model, $Y_{t} = T_{t} S_{t} \varepsilon_{t}$, the forecasting equation is $\hat{Y}_{t} = \hat{T}_{t} \hat{S}_{t}$ where $\hat{T} = 910,099 + 17,509t$; *t* = 1, 2, … where 1 represents January of 2008 and the seasonal index is shown in Table 1. The number of foreign tourists arriving to Thailand is higher than usual during December–March, especially the New Year celebration, that is, there will be the increase in the number of tourists, representing 16%, 13%, 10% and 8%, respectively.

**Table 1:** Seasonal index of international tourist arrivals to Thailand

Jan	Feb	Mar	Apr	May	Jun	Jul	Aug	Sep	Oct	Nov	Dec
1.13	1.10	1.08	0.97	0.87	0.90	1.00	1.02	0.85	0.92	1.00	1.16

#### Holt–Winters’ Exponential Smoothing

Examination of the data for the international tourist arrivals from January 2008 to December 2018 in Fig. 2 indicates that trend and seasonal pattern appear to exist. Winters’ three-parameter linear and seasonal exponential smoothing method decomposes the time series into three components: level, trend, and season. The multiplicative model is demonstrated with the smoothing coefficients of level, trend, and seasonality as $\hat{\alpha }$ = 0.762, $\hat{\beta }$ = 0.001, and $\hat{\gamma }$ = 0.710, respectively.

#### Box–Jenkins

**Fig. 3:**
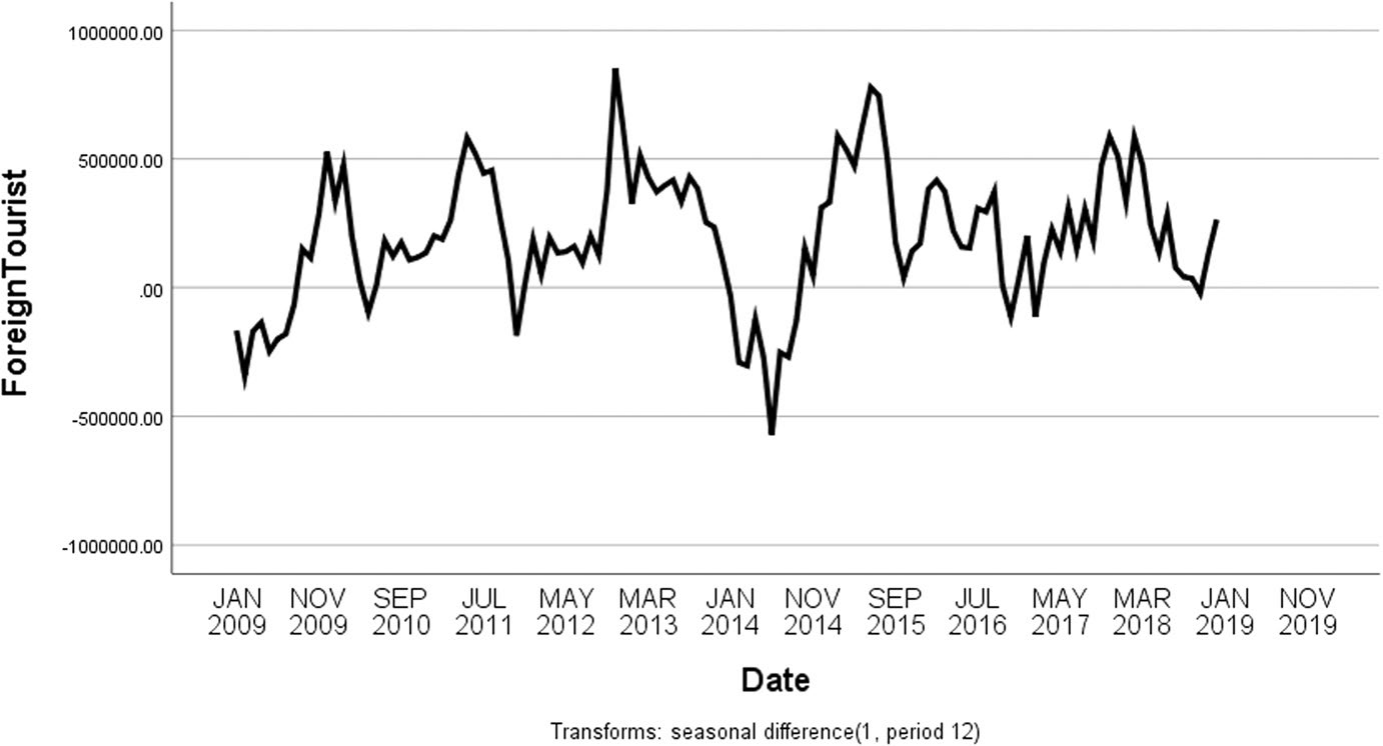
Time series plot for the seasonal differences of international tourist arrivals to Thailand

**Fig. 4:**
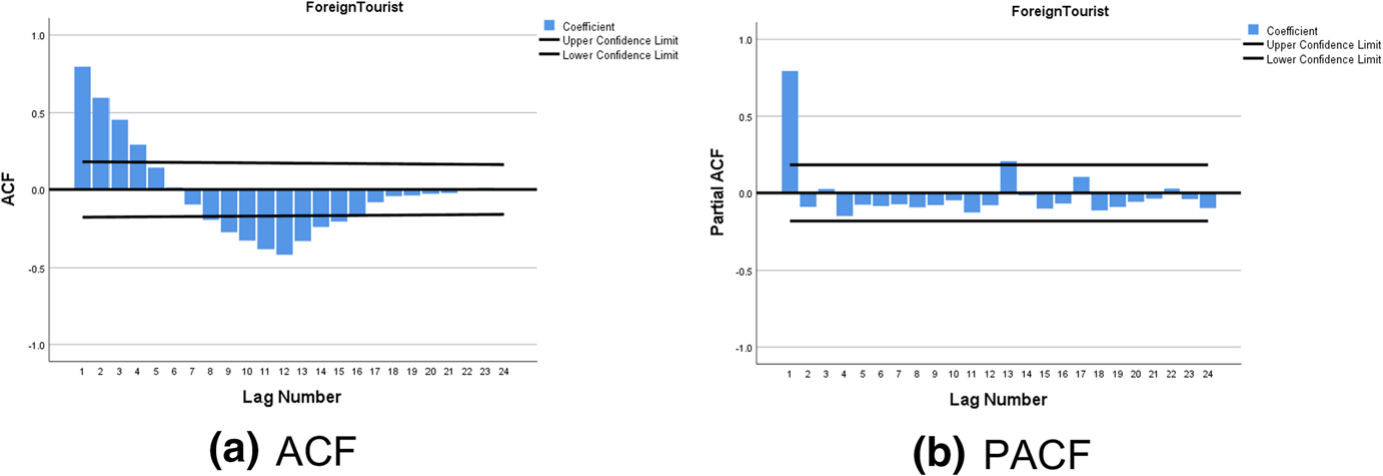
Plots of **a** ACF and **b** PACF for the seasonal differences of international tourist arrivals to Thailand

The monthly international tourist arrivals from January 2008 to December 2018 shows non-stationary pattern because of the upward trend and seasonal components as displayed in Fig. 2. After transforming the time series data with the first season differences, the time series plot in Fig. 3 shows the fluctuation around 200,000. This suggests stationary pattern and there is not necessary to make the first differences. For the model identification, the autocorrelation coefficients trail off to zero gradually as displayed in Fig. 4a, whereas the autocorrelation coefficients drop to zero after the first time lag as shown in Fig. 4b. This suggests AR (p = 1). In addition, the sample partial autocorrelations have significant spike at lag 1 and 13. This behavior suggests an MA (Q = 1) term at the seasonal lag 12.

The suitable model SARIMA(1,0,0)(0,1,1)_12_ in the form of backward operator is

$$( {1 - B^{12} } )( {1 - \phi_{1} B} )Y_{t} = \theta_{0} + ( {1 - \theta_{12} B^{12} } )\varepsilon_{t}$$

or it can be rewritten as

$$Y_{t} = \theta_{0} + \phi_{1} ( {Y_{t - 1} - Y_{t - 13} } ) + Y_{t - 12} - \theta_{12} e_{t - 12} + \varepsilon_{t}$$

with parameter estimates in Table 2. Hence, the forecasting equation is

$$\hat{Y}_{t} = 206,864.98 + 0.774( {Y_{t - 1} - Y_{t - 13} } ) + Y_{t - 12} - 0.636e_{t - 12} .$$

**Table 2:** Estimated parameters for SARIMA(1,0,0)(0,1,1)_12_

Parameter	Estimate	s.e.	*t* test statistic	p-value
$\theta_{0}$	206,864.98	23,340.63	8.863	0.000
$\phi_{1}$	0.774	0.061	12.616	0.000
$\theta_{12}$	0.636	0.096	6.643	0.000

**Fig. 5:**
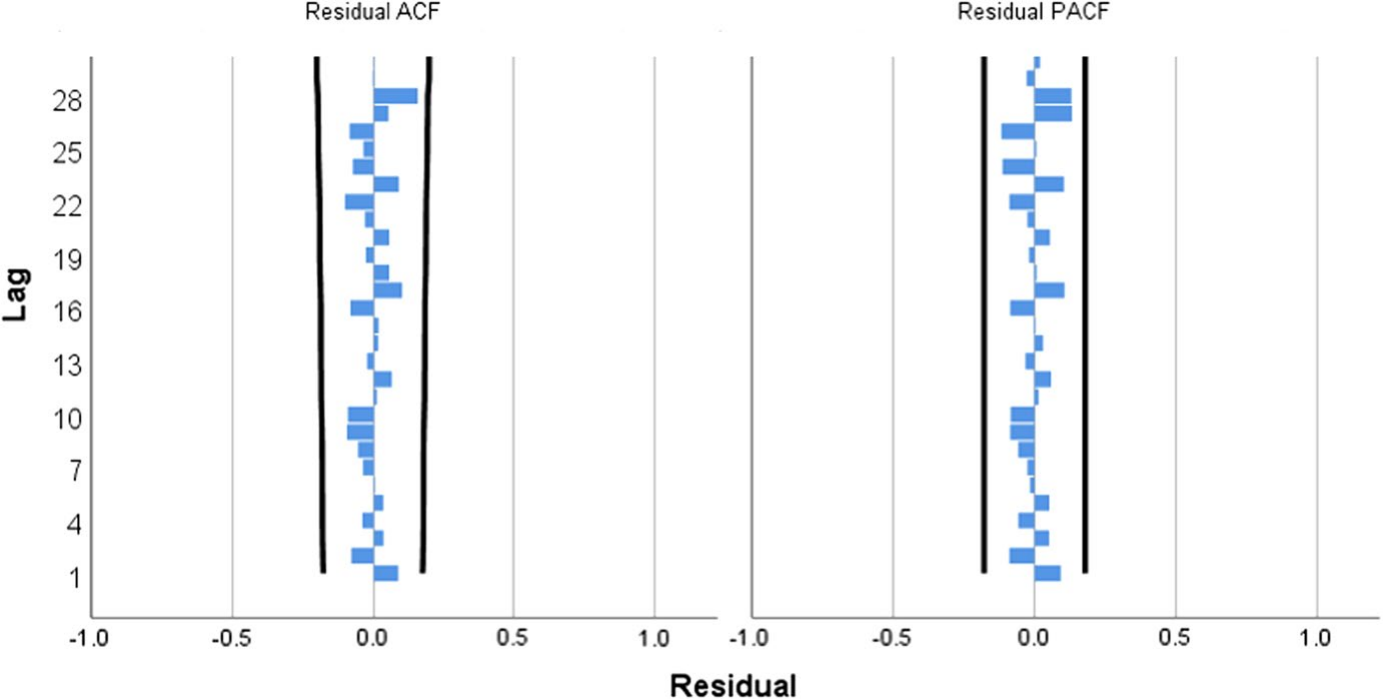
Plots of ACF and PACF for the residuals of SARIMA(1,0,0)(0,1,1)_12_

**Fig. 6:**
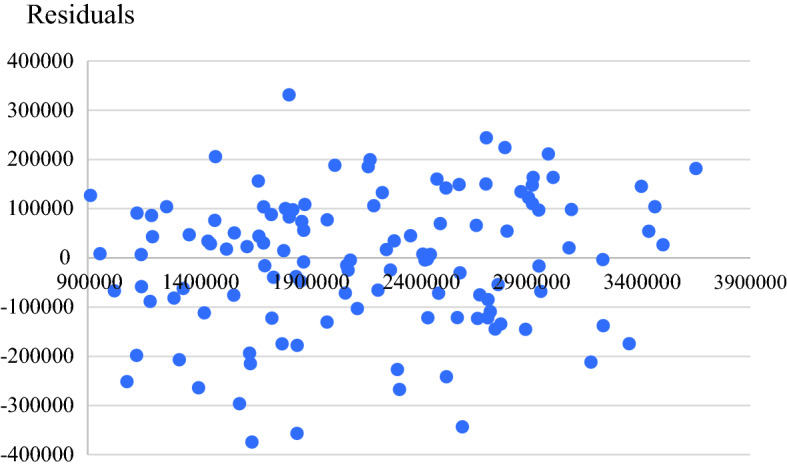
Plots of residuals vs forecast values of international tourist arrivals to Thailand

The model adequacy is done accordingly to the plots of ACF and PACF for the residuals of SARIMA(1,0,0)(0,1,1)_12_ as shown in Fig. 5. These plots satisfy the model assumption and reveal that the residuals are independent because there is no lag of ACF and PACF falling outside the 95% confidence interval. Also, Fig. 6 reveals equal scatter of error terms.

### Model Comparison

According to the criteria of the performance comparison on the mean absolute percentage error and the root mean square error of the three forecasting approaches: decomposition, Holt–Winters and Box–Jenkins, Table 3 shows that the multiplicative Holt–Winters model provides the least values of MAPE and RMSE.

**Table 3:** Comparison of model performance

Method	MAPE (%)	RMSE (people)
Decomposition	6.31	146,491.24
Holt–Winters	5.61	134,694.82
Box–Jenkins	5.88	138,124.91

### Suitable Model for Forecasting and Forecasting Period

**Fig. 7:**
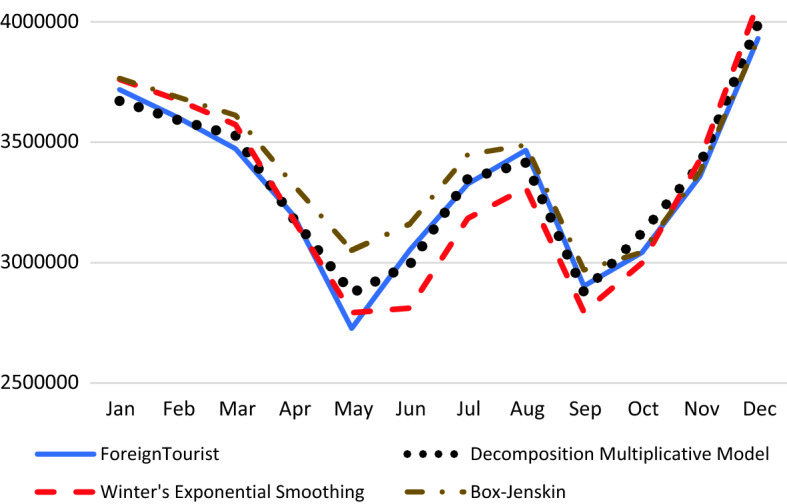
Actual and forecast values of international tourist arrivals to Thailand in 2019

The forecasting values of the monthly international tourist arrivals to Thailand in 2019 are computed based on the forecasting models in Sect. 4.1. Figure 7 displays the comparison of the actual values and the forecasting values of the multiplicative model of decomposition and Holt–Winters and the Box–Jenkins model of SARIMA(1,0,0)(0,1,1)_12_. For the performance of forecasting, Table 4 shows that the multiplicative decomposition method performs the best for short-term forecast.

**Table 4:** Comparison of forecasting performance

Method	MAPE (%)			RMSE (people)		
	3 months	6 months	12 months	3 months	6 months	12 months
Decomposition	1.04	1.81	1.62	42,054.29	71,285.35	62,522.32
Holt–Winter	2.01	2.81	3.05	75,291.41	115,287.00	116,954.59
Box–Jenkins	2.54	4.51	2.86	97,749.53	164,327.05	123,202.81

## Conclusions and Discussion

The demonstration of the classical forecasting techniques and algorithms which are decomposition, Holt–Winters and Box–Jenkins are successfully done using SPSS. The time series of the international tourist arrivals of the year before the Covid-19 pandemic, 2008–2019, are applied. The performances of the model are separated into model and forecasting based on the criterial of the lowest of MAPE and RMSE. For the model comparison, the multiplicative Holt–Winters’ exponential smoothing performs the best followed by the Box–Jenkins, SARIMA(1,0,0)(0,1,1)_12_, and the multiplicative decomposition, respectively. In the view of forecasting performance, the short-, medium-, and long-term forecasts are focused. The multiplicative decomposition is superior for all terms of forecasting followed by Holt–Winters and Box–Jenkins. This point shows that the best model does not always support the superior forecasting.

**Fig. 8:**
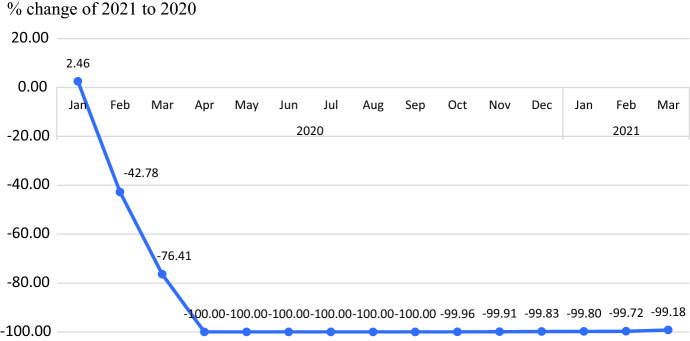
Percentage change of international tourist arrivals to Thailand of 2021 to 2020

The principle of time series forecasting is that the forecast model is created by the pattern or the behavior of the past data. This is the main reason of selecting the time series before the emergence of corona virus pandemic. The limitation of this study is that the forecasting models are valid only for Thailand during the specific period of time. As a result, the number of foreign tourists has changed greatly since February 2020 [1] (see Fig. 8) and more importantly this affects the component of time series data. This severely affects tourism revenue which is the major contributor to the GDP and economics of Thailand and exactly all over the world. The government stakeholders including the private sectors should find policies or measures whether the campaign of getting the Covid-19 vaccine shots to build up the immune response, or the epidemic prevention measures, or the policy of maintaining the tourist attractions to encourage foreign tourists to return to Thailand under normal conditions.

## Abbreviations

MAPE: Mean absolute percentage error
RMSE: Root mean square error
TAT: Tourism Authority of Thailand
UNWTO: World Tourism Organization
CAAT: Civil Aviation Authority of Thailand
ARIMA: Autoregressive integrated moving average
SARIMA: Seasonal autoregressive integrated moving average
ANN: Artificial neural network
ACF: Autocorrelation function
PACF: Partial autocorrelation function
